# Dextran Sulphate Sodium Acute Colitis Rat Model: A Suitable Tool for Advancing Our Understanding of Immune and Microbial Mechanisms in the Pathogenesis of Inflammatory Bowel Disease

**DOI:** 10.3390/vetsci9050238

**Published:** 2022-05-16

**Authors:** Petra Adamkova, Petra Hradicka, Helena Kupcova Skalnikova, Veronika Cizkova, Petr Vodicka, Silvia Farkasova Iannaccone, Monika Kassayova, Sona Gancarcikova, Vlasta Demeckova

**Affiliations:** 1 Faculty of Science, Institute of Biology and Ecology, Pavol Jozef Safarik University in Kosice, 041 54 Kosice, Slovakia; petra.adamkova@student.upjs.sk (P.A.); p.hradicka@gmail.com (P.H.); monika.kassayova@upjs.sk (M.K.); 2 Institute of Animal Physiology and Genetics of the Czech Academy of Sciences, 277 21 Libechov, Czech Republic; skalnikova@iapg.cas.cz (H.K.S.); cizkovav@iapg.cas.cz (V.C.); vodicka@iapg.cas.cz (P.V.); 3 Department of Forensic Medicine, Faculty of Medicine, Pavol Jozef Safarik University in Kosice, 040 11 Kosice, Slovakia; silvia.farkasova.iannaccone@upjs.sk; 4 Department of Microbiology and Immunology, University of Veterinary Medicine and Pharmacy in Kosice, 041 81 Kosice, Slovakia; sona.gancarcikova@uvlf.sk

**Keywords:** DSS-induced colitis, faecal and caecal microbiota, colon cytokines

## Abstract

Inflammatory bowel disease (IBD) is a group of disorders causing inflammation in the digestive tract. Recent data suggest that dysbiosis may play a pivotal role in the IBD pathogenesis. As microbiome-based therapeutics that modulate the gut ecology have been proposed as a novel strategy for preventing IBD, the aim of presenting study was to evaluate the dextran sulphate sodium (DSS) rat model mainly in terms of microbial shifts to confirm its suitability for dysbiosis study in IBD. Acute colitis was induced using 5% DSS solution for seven days and rats were euthanized five days after DSS removal. The faecal/caecal microbiota was analyzed by next generation sequencing. Disease activity index (DAI) score was evaluated daily. Blood and colon tissue immunophenotyping was assessed by flow cytometry and histological, haematological, and biochemical parameters were also evaluated. The colitis induction was reflected in a significantly higher DAI score and changes in all parameters measured. This study demonstrated significant shifts in the colitis-related microbial species after colitis induction. The characteristic inflammation-associated microbiota could be detected even after a five day-recovery period. Moreover, the DSS-model might contribute to an understanding of the effect of different treatments on extraintestinal organ impairments. The observation that certain bacterial species in the gut microbiota are associated with colitis raises the question of whether these organisms are contributors to, or a consequence of the disease. Despite some limitations, we confirmed the suitability of DSS-induced colitis model to monitor microbial changes during acute colitis, in order to test attractive new microbiome-based therapies.

## 1. Introduction

Inflammatory bowel disease (IBD) is a term for two conditions Crohn’s disease (CD) and ulcerative colitis (UC) that are characterized by chronic non-infectious inflammation of the gastrointestinal tract. Between 1990 and 2017, the number of individuals with IBD increased from 3.7 million to more than 6.8 million, an increase of 85.1% in the global prevalence of IBD [1]. The exact aetiology of IBD is not well-known, but there are several factors affecting the development of this disease, which include environmental impact, microbiome dysbiosis, a change in the immune system, and genetic variations [2]. IBD can cause a variety of symptoms, both in and out of the gut. The extraintestinal complications of IBD include arthritis, anaemia, diseases of bones, eyes, skin, kidneys, and liver. These complications can adversely affect all aspects of an individual’s life [3]. Considering all these symptoms and complications, it is important to understand the complex interactions and to develop new targeted therapies of clinical interest. Therefore, animal models of IBD represent contribute tools, which allow manipulations and interventions that are not possible with human studies [4].

The most widely used rodent model of colitis employs dextran sulphate sodium (DSS) mainly due to the reliable induction of gut inflammation, good reproducibility, and several correlations with human IBD [5,6]. In rats and mice, the result of chemically-induced colitis is dependent on the genetic background and the inflammation occurs predominantly in the colon [7]. The induction of colitis by the oral DSS administration is more effective in Sprague Dawley (SD) rats while Wistar rats are more sensitive to TNBS (2,4,6-trinitrobenzene sulphonic acid) which significantly correlates with clinical and morphological features of CD [4,8]. DSS is directly toxic to the colonic epithelium and its short-term administration leads to a very reproducible acute colonic inflammation [9,10]. Depending on the concentration, the duration and frequency of DSS administration, the animals may develop an acute or chronic form of colitis [5]. Acute colitis is usually induced by 4.5–6.0% *w*/*v* DSS (molecular weight 35–55 KD) for 6–10 days [8,11,12,13]. The optimal protocol for inducing the acute phase is 5% *w*/*v* DSS (40,000 g/Mol) for seven days [14,15,16]. The recent results indicate that sulphate groups of the DSS molecules destabilize the mucus layers and make it more permeable to bacteria [17]. Therefore, the DSS model is not simply a model of toxicity, but also a barrier dysfunction model that encompasses mucus loss and the eventual bacterial penetration [18].

Recent clinical and experimental data suggest dysbiosis may play a pivotal role in the pathogenesis of IBD [19]. Moreover, medication for IBD also affects the composition of the gut microbiota [20,21]. As microbiome-based therapeutics that modulate the gut ecology have been proposed as a novel strategy for preventing or alleviating IBD, the aim of the presented work was to evaluate the acute DSS rat model mainly in terms of microbial shifts in order to confirm its suitability for animal dysbiosis study in IBD. Experimental models of IBD could be useful tools to improve the understanding of the mechanistic relations of gut microbiota with IBD pathogenesis.

## 2. Materials and Methods

### 2.1. Experimental Design

Sprague Dawley rats four-weeks old (male, *n* = 22) (Velaz, Prague, Czech Republic) were randomly divided into DSS (*n* = 16) and the healthy control (C) (*n* = 6) groups. Animals were adapted to standard vivarium conditions as previously described [22]. Rats were fed with standard diet (Altromin 1324, Lage, Germany) and had free access to deionized water. Experimental colitis was induced in five-weeks old animals with 5% DSS (40 KD; TdB Consultancy AB, Uppsala, Sweden) dissolved in deionized water given ad libitum for seven days [23]. The rats were euthanized five days after DSS removal to study the progression of acute phase or recovery. Healthy control animals received deionized water only. The body weight gain and food consumption were recorded on a daily basis. The food efficiency ratio percentage (FER) was calculated as body-weight gain in grams divided by food intake in grams multiplied by 100. All animals from both groups were sacrificed by rapid decapitation in anaesthesia. To evaluate the severity of colitis, a disease activity index (DAI) score was determined on a daily basis. The DAI is the combined score of weight loss, stool consistency, and haematochezia. Scores were defined as follows [24]: weight loss: 0 (no loss), 1 (5–10%), 2 (11–15%), 3 (16–20%), and 4 (>20%); stool consistency: 0 (normal), 2 (loose stool), and 4 (diarrhoea); and haematochezia: 0 (absence), and 4 (presence).

### 2.2. Histopathological Analysis and Colon Length

The histopathological analyses were carried out as previously described [22] and the colonic epithelial damage was evaluated referring to the method of Katakura et al. [25].

### 2.3. Blood and Serum Analysis

The basic haematological parameters were determined at three time points: before induction of colitis (day one), at the end of the DSS administration (day seven) and at the end of the experiment (day 13). Blood from the lateral tail vein was collected into K_2_EDTA plastic micropipettes (Boule Medicals AB, Spånga, Sweden) and analysed by blood haematological analyser EXIGO H400 (Boule Medical AB, Spånga, Sweden). The samples for determination of biochemical parameters were obtained during decapitation. Briefly, blood was collected into K_3_EDTA-containing tubes for plasma sampling and serum gel tubes (Sarstedt, Nümbrecht, Germany). K_3_EDTA blood samples were centrifuged at 2400 g at 4 °C for 15 min., and plasma was collected, stored at −80 °C, and later analysed by rat CRP (C-reactive protein) Simplex ProcartaPlex kit (Invitrogen, Paris, France). Blood for serum collection was allowed to clot for at least 30 min. and subsequently tubes were centrifuged at 2400× *g* at 4 °C for 15 min. Serum samples were stored at −20 °C. The biochemical analyses of blood serum were quantified using the Beckman Coulter AU480 (Beckman Coulter, Indianapolis, USA).

### 2.4. Multiplex Cytokine/Chemokine Profiling

Approximately 300 mg of colon tissue were collected into cryovials and stored at −80° C. After tissue homogenization, the concentrations of cytokines and chemokines were measured using a commercial Luminex immunoassay kit (Cytokine & Chemokine 22-Plex Rat ProcartaPlex™ Panel, Invitrogen, Paris, France) according to the manufacturer’s instructions. All analyses were performed using the Luminex^®^ 200™ analyzer (Luminex Corporation, Austin, TX, USA) with the xPonent^®^ Software (Luminex Corporation, Austin, TX, USA) as previously described [26].

### 2.5. Immunophenotyping by Flow Cytometry 

Colon tissue samples for flow cytometric analyses were processed according to the protocol of Bayne and Vonderheide [27]. The tissue dissociation enzyme solution was prepared in RPMI-1640 (Gibco, Massachusetts, Grand Island, NY, USA) and contained 0.25 mg/mL hyaluronidase, 1.5 mg/mL collagenase IIa and 1 mg/mL collagenase IV (Worthington Biochemical Corporation, Lakewood, NJ, USA). Viability and cellularity were determined by Guava Muse Cell Analyzer (Luminex, Austin, TX, USA) using the Muse^®^ Count & Viability Kit (Luminex, Austin, TX, USA). Approximately 1.0 × 10^6^ cells were centrifuged at 1200 rpm for 5 min., the supernatant was discarded and 100 μL of stain buffer (BD Pharmingen™, San Diego, CA, USA) were added. The cell suspension was stained by 1 μL of each antibody for surface markers (BD Bioscience, San Diego, CA, USA) (Table 1) for 15 min. at 4 °C in the dark. After incubation, the cells were stained with propidium iodide (Sigma-Aldrich, St. Louis, MO, USA).

The blood was collected into K_3_EDTA-containing tubes (Sarstedt, Nümbrecht, Germany). Blood samples were stained by antibodies (Table 1) for 15 min. at 4 °C in the dark. Red blood cells were lysed by adding 1 mL of FACS Lysing solution^TM^ (BD Biosciences, San Diego, CA, USA) for 7 min. in the dark at room temperature. The samples were then centrifuged at 1200 rpm for 5 min., supernatant was discarded, and stain buffer was added.

The analyses of tissue and blood samples were performed on BD FACSAria SORP II (Amersham Biosciences, Piscataway, NJ, USA), and leukocytes/live cells were acquired for analysis. The data were analysed in FlowJo software (TreeStar Inc., Ashland, OR, USA). Representative images of the gating strategy of the tissue and blood are shown in Supplementary Material (Figures S1 and S2).

### 2.6. NGS Analysis of Faecal Samples

Faecal samples of rat faeces were collected at three time-points: before induction of colitis, at the end of DSS administration and at the end of the experiment. Caecal content was collected on the day of sacrifice. The samples were placed in −80 °C freezer and subsequent processing for next generation sequencing (NGS) analysis (Novogene Europe, Cambridge, UK) was performed according to Adamkova et al. [22].

### 2.7. Statistical Analysis

Statistical analyses related to the microbiome were performed in Novogene Europe (Cambridge, UK) using QIIME, LEfSe, Flash, R, RDP, Blast (ITS), PyNast Bioinformatics software. All other statistical analyses were performed using Minitab software version 16 (Minitab Inc., 2013, State College, PA, USA). Statistical differences within the group were analysed by a paired T-test if normally distributed or by the Wilcoxon significance test for nonparametric data. Statistical differences between groups (*p* < 0.05) were analysed using two sample T-test if normally distributed or the Mann-Whitney test for nonparametric data. 

## 3. Results

### 3.1. Disease Severity

UC severity was evaluated based on factors such as disease activity index (DAI) and colon-related features. Even though no significant difference in average food intake was observed between experimental groups (Table 2), the DSS group exhibited a significant reduction in body weight gain (*p* < 0.05) compared to the healthy animals which was reflected in a significant decrease (*p* < 0.01) of FER.

**Figure 1:**
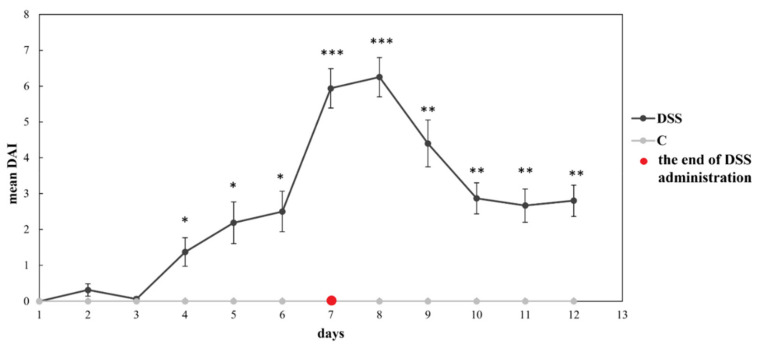
Disease activity index (DAI). Data are expressed as mean ± SEM (* *p* < 0.05, ** *p* < 0.01 and *** *p* < 0.001); (*n* = 16/DSS group; and *n* = 6/C group); DSS, induced colitis; C, healthy control.

The clinical signs of DSS-induced colitis in rats were assessed by the DAI providing indirect information on the inflammation severity which can correlate with the damage to the intestinal mucosa. The DAI, calculated based on the percentage of body weight decrease, diarrhoea, and bloody faeces, increased from day three in DSS-treated rats (Figure 1) with the peak on the day seven and eight. The DAI score remained close to 0 in the non-colitis control group during the whole experimental period (no diarrhoea or rectal bleeding was observed).

**Figure 2:**
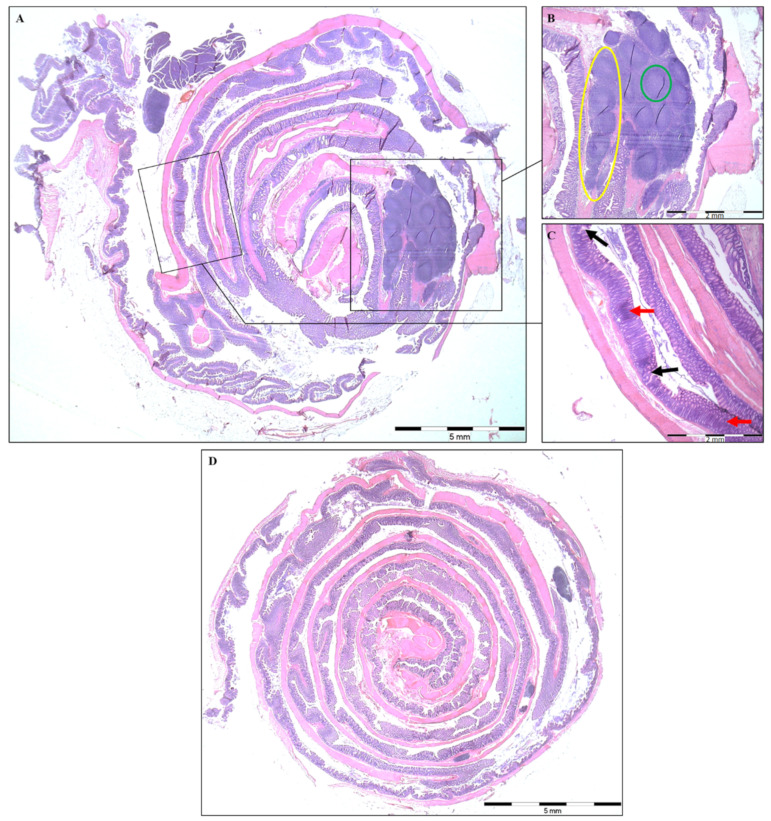
Histopathological features present in the colon of experimental animals. Representative Swiss-rolls of colon (H&E staining) from DSS-treated rats (**A**) and healthy control animals (**D**). Control group maintained normal colon morphology, whereas the DSS group shows numerous lymphoid aggregates (**B**, yellow circle) and lymphoid follicles (**B**, green circle), the multifocal areas of the mucosal erosions with the loss of epithelial cells (**C**, black arrow) and inflammatory cell infiltration (**C**, red arrow).

**Figure 3:**
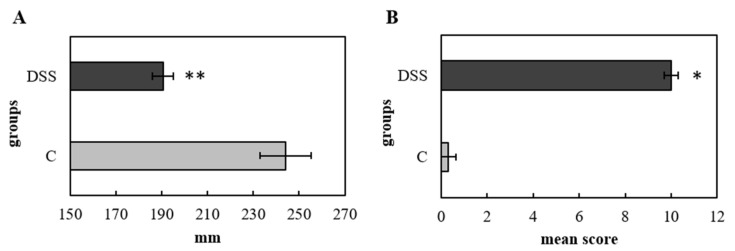
Histopathological changes reflected in the length of the colon (**A**) and histopathological score (**B**). Data are expressed as mean ± SEM (*n* = 16/DSS group; *n* = 6/C group), where a * denotes for *p* < 0.05 and ** for *p* < 0.01. DSS, induced colitis; C, healthy control.

Histological analysis of colonic mucosa showed multifocal areas of erosion as well as regions of more severe (50–90%) or complete crypt loss in the DSS group (Figure 2A). Large ulcers (≥10 crypt widths) as well as infiltration with inflammatory cells in the mucosa and submucosa (Figure 2C), and numerous lymphoid aggregates and follicles (Figure 2B) were observed in the DSS group. The colonic mucosa of the control group remained intact, with normally differentiated epithelial cells and without signs of active inflammation or an increased density of immune cells (Figure 2D). Overall, the histopathological score was significantly increased in the DSS group (*p* < 0.05) (Figure 3B), which was also reflected in decreased colon length (*p* < 0.01) (Figure 3A). Evaluation of the lymphoid aggregates showed a significantly higher mean length (*p* < 0.001) and width (*p* < 0.01) in the DSS group in comparison to control animals (Table 3). These results also indicated that colitis was successfully induced in rats.

### 3.2. Biochemical, Haematological and Plasma CRP Analyses

**Figure 4:**
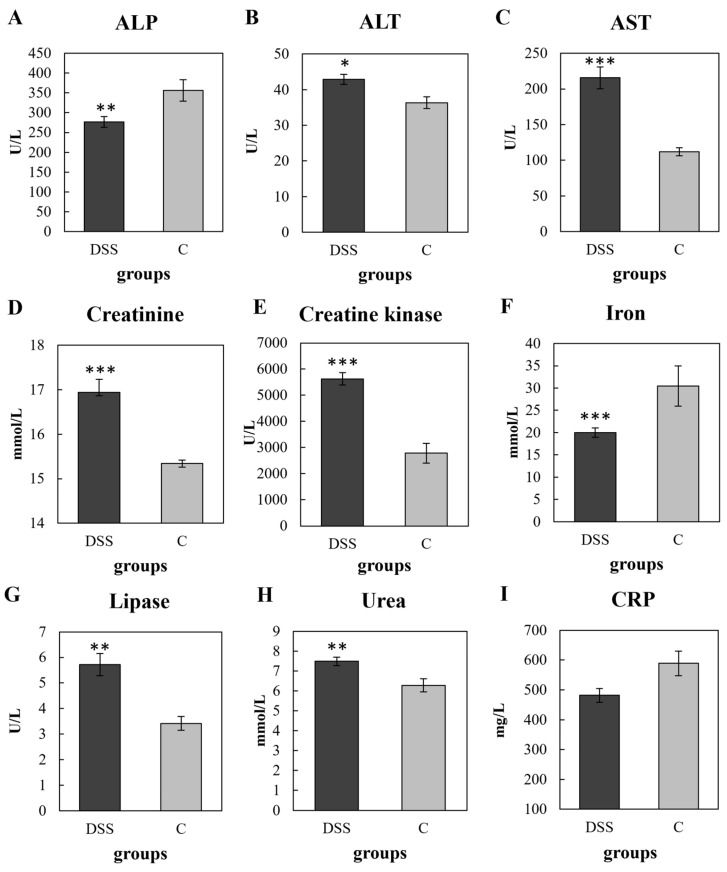
Serum levels of biochemical parameters in animals with DSS-induced colitis and controls. Concentration of ALP (**A**); ALT (**B**); AST (**C**); creatinine (**D**); creatine kinase (**E**); iron (**F**); lipase (**G**) urea (**H**) and CRP (**I**). Data are expressed as mean ± SEM (*n* = 16/DSS group; *n* = 6/C group); (* *p* < 0.05; ** *p* < 0.01; *** *p* < 0.001). DSS, induced colitis; C, healthy control. ALP, alkaline phosphatase; ALT, alanine aminotransferase; AST, aspartate aminotransferase; CRP, C-reactive protein.

Serum biochemical analyses were performed at the end of the experiment to detect hepatic, renal, and pancreatic function. As shown in Figure 4, DSS administration significantly increased the levels of ALT (*p* < 0.05), AST (*p* < 0.001), creatinine (*p* < 0.001), creatine kinase (*p* < 0.001), lipase (*p* < 0.01) and urea (*p* < 0.01), while the ALP (*p* < 0.01) and iron (*p* < 0.001) were significantly decreased. Plasma CRP levels are known to increase dramatically in response to inflammation [28], however, in this study, we did not observe a difference in CRP level in the DSS group compared to the control group (Figure 4I). The haematological parameters of the experimental groups are represented in Table 4. At the end of the DSS administration (2nd collection time-point) a significant increase in the total leukocyte (*p* < 0.001), lymphocyte (*p* < 0.001), monocyte (*p* < 0.01) and granulocyte (*p* < 0.001) counts were observed in the DSS group compared to initial values. The platelet to lymphocyte ratio and the lymphocyte to monocyte ratio significantly decreased (*p* < 0.001) while the granulocytes to lymphocyte ratio increased (*p* < 0.001) in response to DSS administration. Compared to the control group, increased counts (*p* < 0.05) of total leukocytes, lymphocytes, monocytes, and granulocytes were observed at the end of colitis induction. Moreover, a decrease in red blood cell count in the DSS group was observed at the end of the experiment (*p* < 0.05).

### 3.3. Multiplex Cytokine/Chemokine Profiling

**Figure 5:**
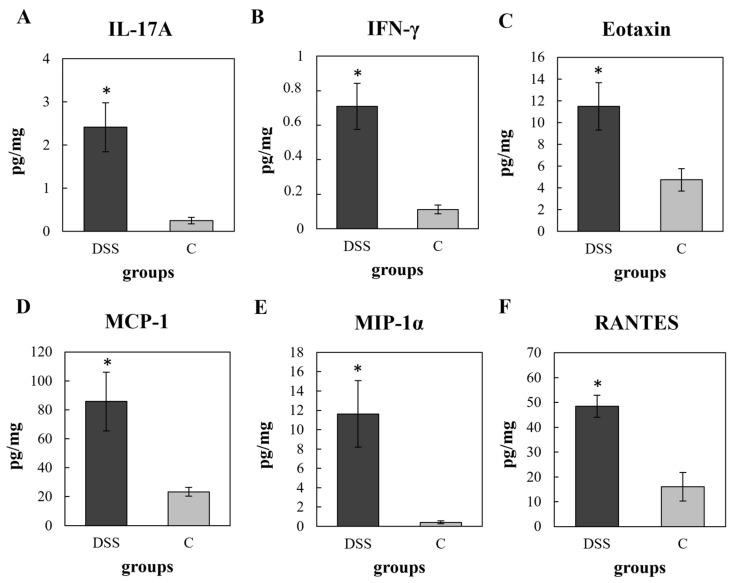
Significantly increased levels of pro-inflammatory cytokines: IL-17A (**A**); IFN-γ (**B**) and chemokines: eotaxin (**C**); MCP-1 (**D**); MIP-1α (**E**) and RANTES (**F**) in the colon tissue of DSS group. Data are expressed as mean ± SEM (*n* = 16/DSS group; *n* = 6/C group); (* *p* < 0.05). DSS, induced colitis; C, healthy control. MCP-1, monocyte chemoattractant protein-1; MIP-1α, macrophage inflammatory protein 1-alpha; RANTES, regulated on activation, normal T-cell expressed and secreted.

The evaluation of colon tissue cytokines showed elevated levels of pro-inflammatory cytokines such as IL-1α, IL-1β, IL-2, IL-12p70 (IL-12), G-CSF, and TNF-α, and a decrease in the anti-inflammatory cytokine IL-13 in the DSS group but not reaching statistical significance (Table S1 in Supplementary Material). On the other hand, the cytokines related to progression of acute colitis, such as IL-17A and IFN-γ, were significantly increased in the DSS group (*p* < 0.05) (Figure 5A,B). DSS treatment also induced significant changes in the levels of the key chemokines that regulate migration and infiltration of monocytes/macrophages—MCP-1 and MIP-1α (*p* < 0.05) (Figure 5D,E), the chemokine responsible for attracting immune cells from the peripheral blood to the sites of inflammation—RANTES (*p* < 0.05) (Figure 5F) and in eotaxin (*p* < 0.05), which plays an important role in the pathogenesis of colitis (Figure 5C).

### 3.4. Flow Cytometric Analysis

**Figure 6:**
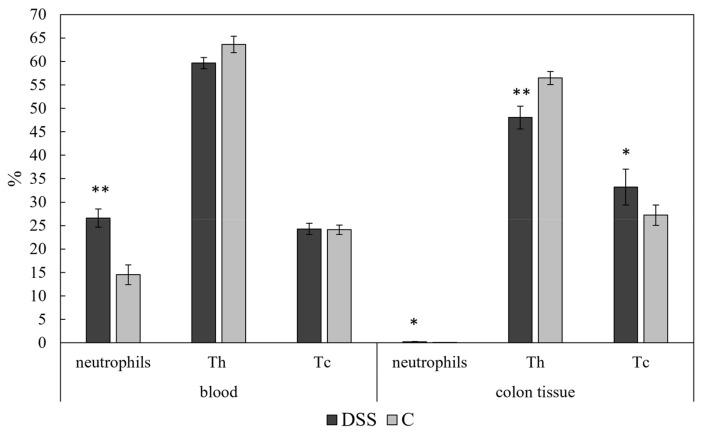
Immunophenotyping of blood and colon tissue immune cells. Data are shown as mean ± SEM (*n* = 16/DSS group; *n* = 6/C group); (* *p* < 0.05; ** *p* < 0.01). DSS, induced colitis; C, healthy control. Th, helper T lymphocytes; Tc, cytotoxic T lymphocytes.

A significant increase in the percentage of neutrophils (CD11b^+^Gr^+^) was evident in the group with acute colitis, both in the blood (*p* < 0.01) and in the colon tissue (*p* < 0.05) (Figure 6). However, no significant differences in the percentage of circulating helper (CD4^+^CD8^−^) and cytotoxic (CD4^−^CD8^+^) T lymphocytes were detected (Figure 6), the proportion of tissue helper T cells significantly decreased in the DSS group (*p* < 0.01), while cytotoxic T lymphocytes were significantly increased in the colon tissue in this group (*p* < 0.05).

### 3.5. Microbiome Analysis of Rat Faecal Samples 

**Figure 7:**
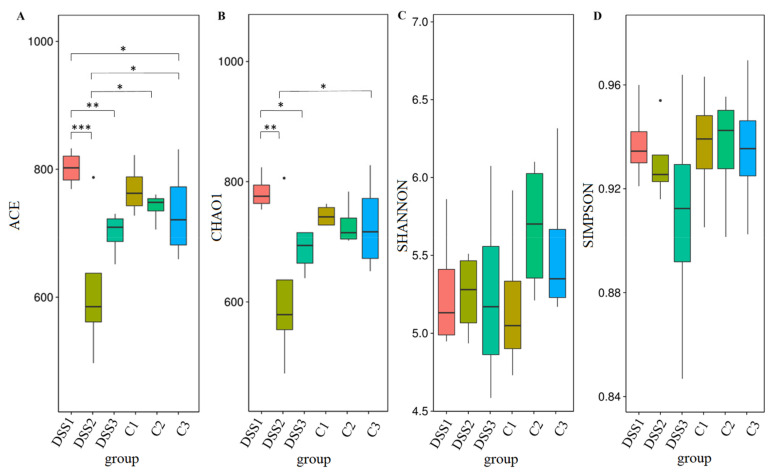
Boxplots of Alpha diversity indices. Numbers next to the group names represent time-points of faecal samples collection: 1, before induction of colitis (day 1); 2, at the end of DSS administration (day 7); 3, at the end of experiment (day 13). DSS, induced colitis; C, healthy control. Wilcoxon significance test: ACE diversity index (**A**): *** *p* < 0.001 for DSS1 vs DSS2, ** *p* < 0.01 for DSS1 vs DSS3; * *p* < 0.05 for DSS1 vs C3, DSS2 vs C2, DSS2 vs C3. Chao1 diversity index (**B**): ** *p* < 0.01 for DSS1 vs DSS2; *p < 0.05 for DSS1 vs DSS3, DSS2 vs C3. Shannon (**C**) and Simpson (**D**) diversity index.

Alpha diversity was quantified by ACE and Chao1 diversity indices, which reflect the Operational Taxonomic Unit (OTU) abundance and by Shannon and Simpson diversity indices, which reflect the diversity of OTU in samples. The ACE and Chao diversity indices (Figure 7A,B) showed a reduction of the OTU abundance in the samples DSS2 (day 7; pACE = 0.0001; pChao = 0.002) and DSS3 (day 13; pACE = 0.0043; pChao = 0.029) compared to the first collection time point (before DSS administration). Statistical testing of the OTU diversity (represented by Simpson and Shannon metrics) showed no differences (Figure 7C,D).

**Figure 8:**
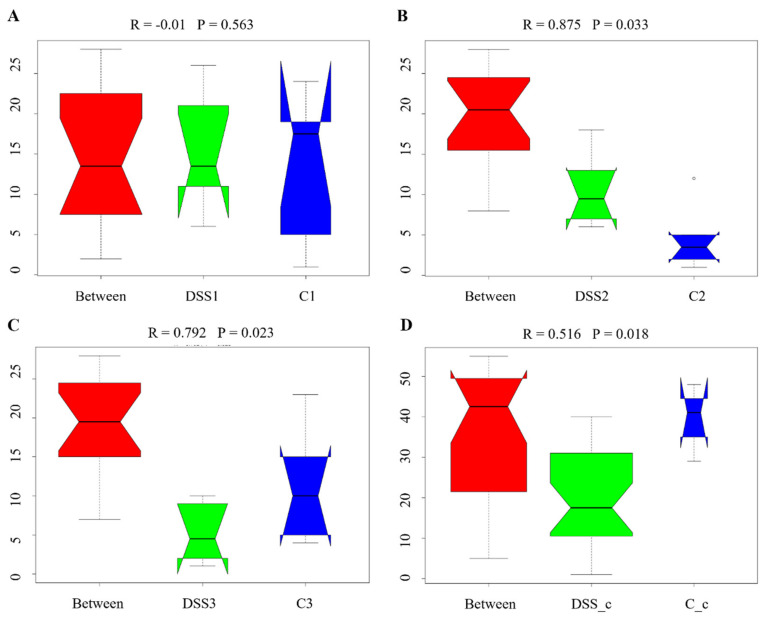
Anosim analysis results. R-value close to 1 shows that intra-group similarities are greater than inter-group similarities. “Between” represents the difference between groups; the greater distance shows the greater difference, and the thickness represents the sample size. Numbers next to the group names represent time-points of faecal samples collection: 1, before induction of colitis (**A**) (day 1); 2, at the end of DSS administration (**B**) (day 7); 3, at the end of experiment (**C**) (day 13); c shows caecal sample collected on the day of sacrifice (**D**). DSS, induced colitis; C, healthy control.

**Figure 9:**
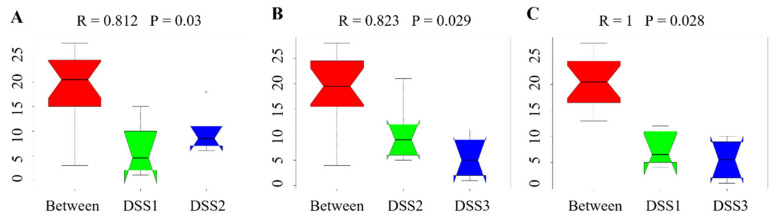
Anosim analysis results within the DSS group. (**A**) day 1 vs day 7; (**B**) day 7 vs day 13; (**C**) day 1 vs day 13. R-value close to 1 shows that intra-group similarities are greater than inter-group similarities. „Between” represents the difference between groups; the greater distance shows the greater difference, and the thickness represents the sample size. Numbers next to the group name represent time-points of faecal samples collection: 1, before induction of colitis (day 1); 2, at the end of DSS administration (day 7); 3, at the end of experiment (day 13). DSS, induced colitis.

Analysis of similarities (ANOSIM) demonstrated the difference in the gut microbiota of colitic and healthy rats (Figure 8). While no significant inter-group differences (R = −0.01) were observed before DSS administration (Figure 8A), DSS treatment induced significant changes (day 7; R = 0.875 for DSS2 vs C2) (Figure 8B) which were preserved until the end of the experiment (day 13; R = 0.792) (Figure 8C). Greater inter-group than intra-group differences were also observed in the caecal samples of the experimental groups (R = 0.516) (Figure 8D). Figure 9 demonstrates ANOSIM analysis within the DSS group. After 7 days of DSS administration, inter-group differences significantly increased (R = 0.812) (Figure 9A) and greater inter-group beta distance was also preserved at the end of experiment (R = 0.823 for DSS2 compared to DSS3) (Figure 9B). Figure 9C shows the highest inter-group distance (R = 1) with statistical significance *p* = 0.028 between the 1st and 3rd collection point.

**Figure 10:**
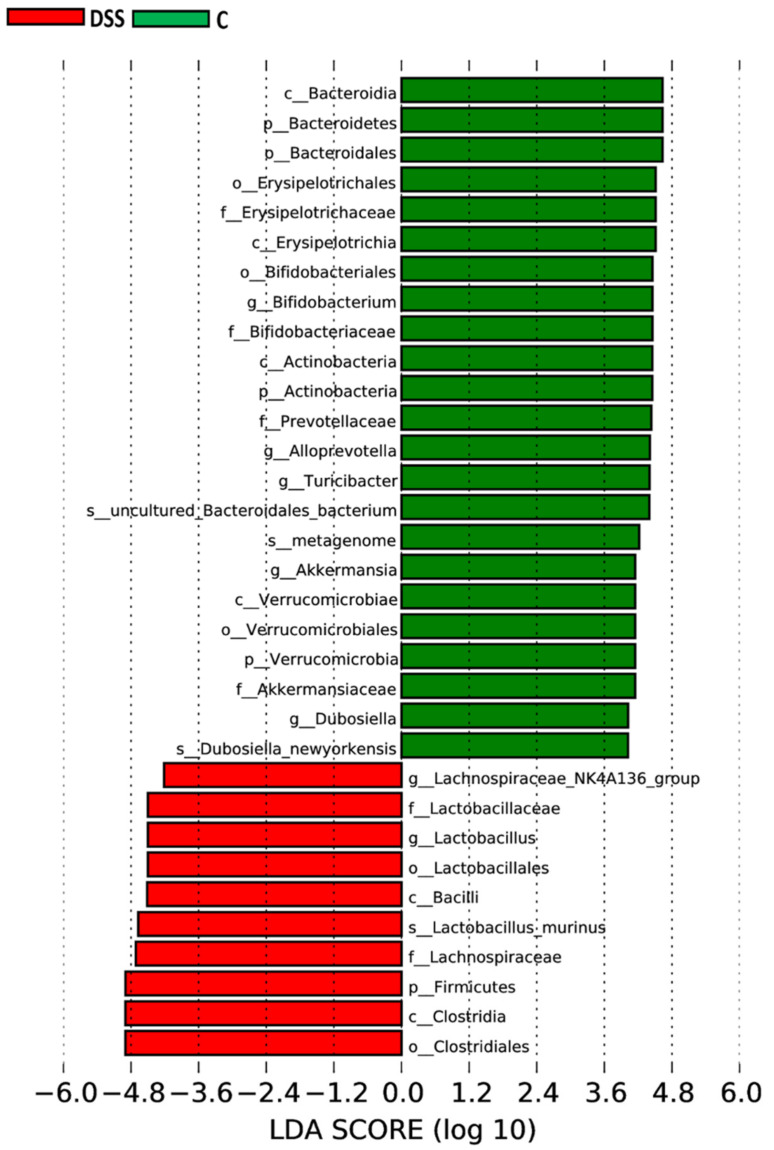
Histogram of LDA scores. Horizontal bars represent the effect size for each taxon. The length of the bar represents the log_10_ transformed LDA score, indicated by vertical dotted lines. *p*-phylum; c, class; o, order; f, family; g, genus and s, species. DSS, induced colitis; C, healthy control.

Figure S3 (in Supplementary Material) and Figure 10 show specific phylotypes that respond to DSS treatment using the linear discriminant analysis (LDA) effect size (LEfSe) algorithm to identify more abundant taxa in the DSS group compared to the group of healthy animals. In total, 33 bacterial taxa were identified to be differentially abundant between the groups, including 4 phyla, 6 classes, 6 orders, 6 families, 7 genera, and 4 species (LDA score > 3.6). The control group was predominantly occupied with phylum Bacteroidetes, class Bacteroidia, and genera *Bifidobacterium*, *Alloprevotella*, *Turicibacter* and *Akkermansia*. The DSS group was mainly dominated by phylum Firmicutes, class Clostridia, family Lachnospiraceae and species *Lactobacillus murinus*.

**Figure 11:**
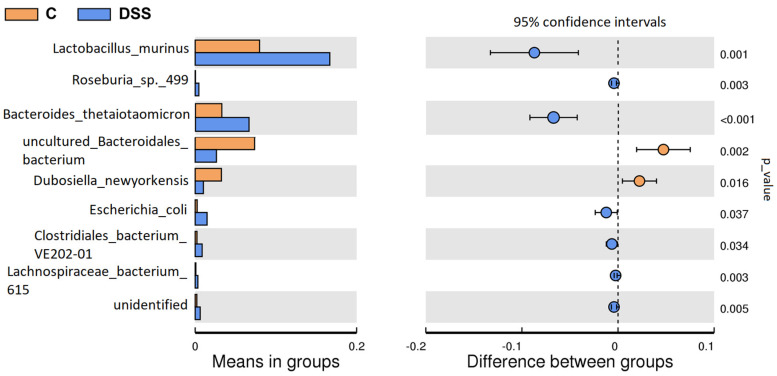
Between-group T-test analysis of faecal samples. DSS, induced colitis; C, healthy control.

The T-test bar plot displays differences in the microbial composition between the control and the DSS-treated group at the species level (Figure 11). *Lactobacillus murinus* (*p* = 0.001), *Bacteroides thetaiotaomicron* (*p* < 0.001), *Escherichia coli* (*p* = 0.037), *Clostridiales bacterium* (*p* = 0.034) and *Lachnospiraceae bacterium* 615 (*p* = 0.003) were significantly increased in the DSS group, while uncultured *Bacteroidales* bacteria (*p* = 0.002) and *Dubosiella newyorkensis* (*p* = 0.016) were significantly decreased.

A Venn diagram showed that 510 OTUs were shared between DSS animals at different time points, and the number of unique OTUs in the first, the second and the third collection point was 235, 81, and 239, respectively (Figure S4 in Supplementary Material). The total richness of all the groups was 1371. After 7 days of DSS administration, 684 OTUs were still shared between DSS-treated animals, however, there were just 551 OTUs shared between 1st and final faecal collection time points.

**Figure 12:**
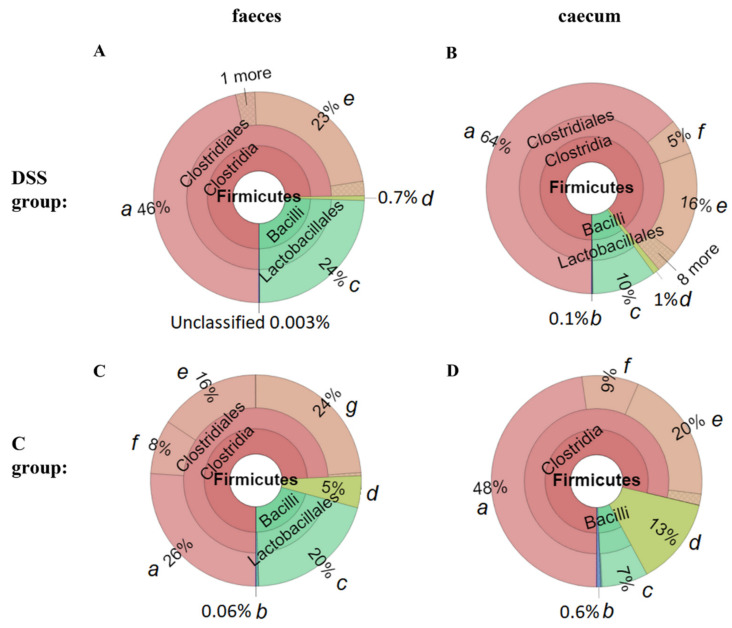
Representative Krona diagrams visually display the difference between faecal samples from DSS (**A**) and C group (**C**) and caecal samples in DSS (**B**) and control group (**D**). a, Lachnospiraceae; b, Selenomonadales; c, Lactobacillaceae; d, Erysipelotrichaceae; e, Ruminococcaceae; f, Peptostreptococcaceae; g, Clostridiaceae_1; DSS, induced colitis; C, healthy control.

**Figure 13:**
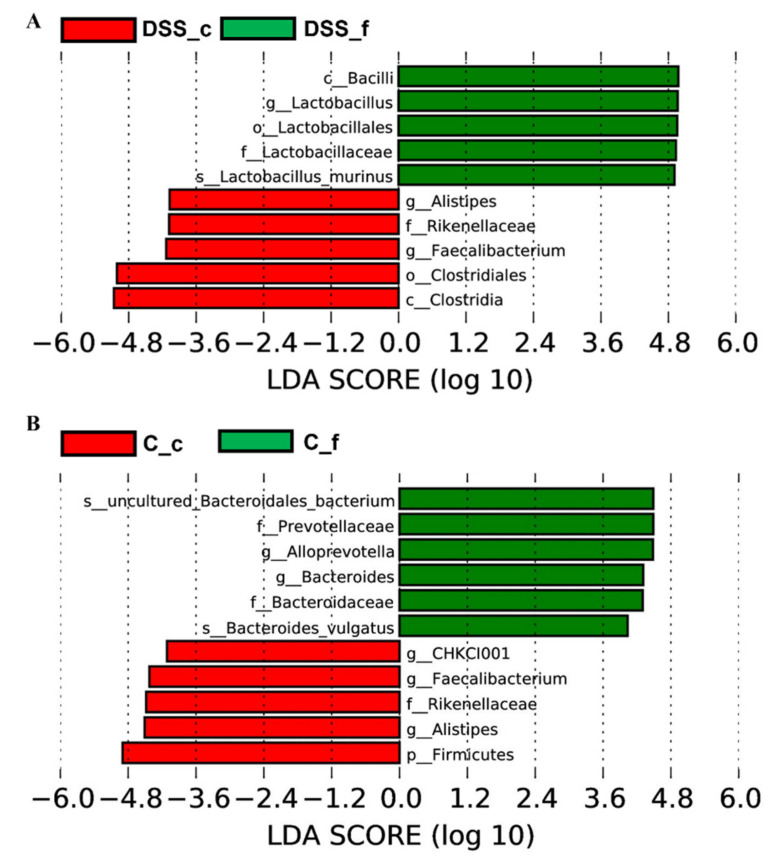
Histogram of LDA scores of caecal (_c) and faecal (_f) samples in DSS (**A**) and control group (**B**). Faecal and caecal samples were collected on the day of sacrifice. p, phylum; c, class; o, order; f, family; g, genus and s, species. DSS, induced colitis; C, healthy control.

Representative Krona diagrams in Figure 12 show abundance of different families within Firmicutes in faeces (Figure 12A,C) and caeca (Figure 12B,D). Lactobacillaceae were more abundantly represented in the faeces of both experimental groups, while Lachnospiraceae and Ruminococcaceae dominated in the caecum. Faecal and caecal samples from the DSS group (Figure 13A) were compared using the linear discriminant analysis (LDA) effect size (LEfSe) algorithm, which shows caecal enrichment in the taxa of Clostridiales. The order Lactobacillales was the more abundant taxa in both groups (Figure 12). Caeca in the control group (Figure 13B) were predominantly occupied with genera *Alistipes* and *Faecalibacterium* in comparison to faeces, where the dominant genera were *Alloprevotella* together with *Bacteroides*.

## 4. Discussion

Rodent models of colitis are generally used to study the pathophysiology of disease and to develop new treatment modalities. Different animal models may reflect human IBD subtypes, however, no single model captures the complexity of human IBD, but each model provides valuable insights into various major aspects of disease, and together they have led to the establishment of generally accepted principles of IBD pathogenesis. Making the choice of which model to use should combine the research question and the IBD subtype to achieve the best outcome. The UC induction using DSS is the most used model in preclinical research studies due to its controllability, simplicity and reproducibility [29]. DSS, exhibits several morphological and pathophysiological features such as superficial ulceration, mucosal damage, production of inflammatory mediators and leukocyte infiltration [4,5,6,30,31]. DSS administration in rats is accompanied with pronounced weight loss (about 5–10% reduction by day five), altered stool consistency leading to diarrhoea and haematochezia [32]. The typical histological changes include mucin and goblet cell depletion, epithelial erosion, ulceration and infiltration of granulocytes into the lamina propria and submucosa [9]. Our results showed that the experimental group with DSS-induced colitis exhibited several symptoms of typical UC histological changes mentioned above, which were reflected in a higher histopathological score, which may be explained by high infiltration of inflammatory immune cells confirmed by flow cytometry of colon tissue. These histological changes were reflected in a higher histopathological score, which could be explained by high infiltration of leukocytes confirmed by flow cytometry of colon tissue.

Neutrophils represent the main leukocytes involved in the inflammation [33]. This study confirmed uncontrolled neutrophil accumulation in the colon tissue, leading to tissue damage and other extraintestinal manifestations. Within the specific immunity, substantial changes in subpopulations of tissue T cells were found in the DSS group where we observed an increase of Tc lymphocytes and a decrease of Th lymphocytes. Our findings are consistent with other studies, which confirmed that reduction of Th lymphocytes with a concomitant increase of cytotoxic T lymphocytes may be considered a marker of active ulcerative colitis [34,35,36]. Tissue-resident Tc lymphocytes are thought to contribute to mucosal damage during disease development [37]. The study with TNBS-induced colitis showed that Tc lymphocytes are responsible for initiation of relapsing colitis in normal immunocompetent mice [38].

Although there are no available blood tests for IBD, different haematological parameters are used to indicate active gut inflammation [39]. Moreover, alterations of leukocyte numbers are recognized early on in IBD [40]. A significant increase of total leukocytes, including lymphocytes, monocytes and granulocytes was present in blood of the DSS group at the end of the DSS administration with a subsequent decrease in RBC count observed at the end of the experiment. These results might be associated with massive injury of the intestinal barrier in the DSS treated animals. Confirmed higher level of neutrophils in peripheral blood was reflected in significantly higher granulocyte to lymphocyte ratio (compared to initial values) which is the most important prognostic factor for UC progression. It is well known that granulocytes play detrimental role in acute phase of colitis [41,42].

The production of cytokines and chemokines by various mucosal cells during intestinal inflammation is an important regulator of neutrophil infiltration (as confirmed by immunophenotyping of colon tissue). For example, increased IFN-γ observed in our study, is considered a major driver of an excessive immune response, leading to massive leukocyte infiltration and mucosal damage [43,44,45]. Moreover, IL-17, which was also significantly elevated in our study, has been shown to be essential for neutrophil recruitment during colitis [46,47]. It was also confirmed, that neutrophils have the ability to produce interleukin-17 [48]. During intestinal inflammation, neutrophils present in the blood sense the chemoattractant gradient produced by resident macrophages, which stimulates their transition into the intestinal tissue. As observed in our study, the number of neutrophils was significantly elevated in the blood as well as the colon tissue in colitic animals. It is known, that neutrophils produce monocyte chemoattractants, therefore creating a vicious circle of inflammation [49]. As reported in the study of Cherfane et al., [50] elevated monocyte and neutrophil counts together with decreased LMR values significantly differentiate between active and quiescent UC.

Significantly increased level of MCP-1 was observed in the group with acute colitis. This finding is in accordance with other studies, where the elevation of MCP-1 was observed in mucosal tissues from patients with CD and UC [51,52,53] and also in experimental models of colitis [54]. MCP-1 is produced by a variety of cells including dendritic cells, fibroblasts, endothelial cells, and macrophages [55]. M1 macrophages produce also other pro-inflammatory cytokines and chemokines, among others eotaxin and MIP-1α, which are considered biomarkers of the M1 macrophage phenotype [56,57]. Moreover, a significantly increased level of MIP-1α in the DSS group suggests the presence of pro-inflammatory M1 macrophages in the colon tissue leading to disease perpetuation and tissue destruction. Our results are consistent with similar studies which confirmed that intestinal macrophages in IBD patients produce more pro-inflammatory cytokines thus promoting/perpetuating the pathological environment [58,59,60,61]. RANTES is another C-C chemokine that promotes the recruitment and activation of inflammatory cells, and which has also a crucial role in the progression from acute to chronic colitis [62]. The increased expression of the chemokine RANTES was observed in patients with ulcerative colitis [63] as well as in the DSS group of this experimental model.

CRP is reported to be the most used marker of inflammatory process [64]. However, the level of CRP in rats is 100 times higher compared with humans [65]. Although different studies used the rat CRP as the inflammatory indicator [66,67,68], in our study, a significant difference of plasma CRP was not observed. It is necessary to point out, that CRP concentration was measured 5 days after cessation of DSS treatment. It was demonstrated that in humans, plasma CRP levels increase from around 1 µg/mL to over 500 µg/mL within 24–72 h of severe tissue damage [69], but when the stimuli ends, CRP values decrease exponentially over 18–20 h [70]. In a study conducted by Mürüvvet and Pinar [71] the CRP concentration almost doubled on the first day in rats with bacterial inflammation, while no increase was determined on other days. Our results are supported by the white blood cell count where a significant increase was only observed immediately at the end of DSS administration, but after the resting period the numbers were approaching the normal values, even still increased. This can be an indicator of a slow resolution of the inflammation during the resting period.

The severity of colon injury was also confirmed by different biochemical parameters, which are linked not only to the intensity of gut inflammation but also to different extraintestinal organ impairments. Clinical and epidemiological evidence suggests that IBD is associated with extraintestinal manifestations (liver, kidneys, pancreas) in 6% to 47% of patients [72].

The liver injury in colitic rats, reported by increased ALT and AST levels, are directly linked to the severity of intestinal damage which suggests that control of liver homeostasis is influenced by the colon condition [73]. Creatinine [74] as well as creatine kinase [75] are reliable indicators of renal function. The concentration of both parameters was significantly increased in DSS-treated animals. The studies using mouse models confirmed renal injury after DSS-administration [76,77]. As renal function decreases, waste products cumulate in the gut epithelium [78], which promotes the colonization of urease-producing bacterial species, that are able to use urea as a source of energy. Moreover, urea was statistically increased in the DSS group, suggesting kidney malfunction, and favouring of ureolytic bacteria. Urease producing bacteria are frequently gram negative Enterobacteriaceae [79] but many strains in the family of Clostridiaceae have also ureolytic ability [80,81]. Several species from these families were also increased at the end of DSS administration in our study. Consequently, the higher level of ammonia is associated with higher intestinal pH, which cause disintegration of colon epithelial barrier [82].

High levels of serum pancreatic enzymes might be associated with extensive and severely active colonic disease [83]. The lipase levels were increased in the DSS group, suggesting either extraintestinal involvement of the pancreas as part of the inflammatory process or leakage of pancreatic enzymes from an inflamed gut [84,85]. The effect of inflammatory mediators and cytokines released from the inflamed gut on the pancreas may also contribute to pancreatic damage and leakage of pancreatic enzymes into the blood [83,86,87]. There are a few reports of elevated lipase in patients with IBD [83,86,88,89]. The study of Ray and van Arsdall reported that during the initial presentation of IBD in their patients, lipase was elevated to more than three times the upper limit of normal [89].

Alkaline phosphatase represents another biochemical parameter which confirmed the presence of an inflammatory condition in chemically induced colitis. Decreased expression of ALP has been demonstrated to be associated with IBD [90,91]. A lower level of ALP is responsible for increased gut permeability, inflammation as well as for gut microbiota changes. In addition to providing a defense against microbes, recent evidence supports that ALP plays a role in determining which bacteria colonize the gut [92]. It was confirmed that ALP knock-out mice faeces contained fewer and less diverse bacteria than the faeces of wild-type mice. Phylogenetic analysis showed that ALP-knock-out mice have more clostridia belonging to the Firmicutes phylum than control mice. Decreased expression of ALP has also been reported in paediatric patients with IBD [93].

Iron deficiency is one of the most common systemic complications of IBD [94]. This study showed an iron deficiency in DSS-treated rats, even thought we did not observe a low level of haemoglobin. These results could be explained by the study of González Alayón et al. [95], where iron deficiency was found in 89.1% of patients with normal haemoglobin levels and was described as the precursor to anaemia. The most important cause of iron deficiency in patients with IBD is increased iron loss due to ongoing gastrointestinal blood loss from the inflamed mucosa. Moreover, iron deficiency has been reported to alter the gut microbiota [96]. Our microbial analyses confirmed the results of an in vitro colonic fermentation study [97] which showed that during very low iron conditions, several taxa including *Bacteroides* were decreased, while members of the *Lactobacillus* and Enterobacteriaceae family were increased. In summary, the biochemical results presented here suggest that the DSS-induced colitis model can provide additional information on the effect of specific treatments on IBD-associated renal, liver and pancreatic malfunction as well as iron deficiency/anaemia. Based on the clinical signs, most of the modern therapeutic approaches manage to control inflammation by applying different immune modulators. Despite we still do not know whether gut dysbiosis is a cause or a consequence of inflammation, modulation of intestinal microbiota might represent a promising approach in UC treatment [98,99]. Therefore, the main aim of our study was to confirm, if a DSS-rat model is an appropriate animal model to test new attractive microbiome-based therapies. The specific intestinal microbiota (mainly species from Bacteroides and clostridia) is directly involved in gut immunity, especially in the regulation of CD4 T-cells, which are responsible for intestinal inflammation [100]. As microbiota diversity is very important for gut ecosystem, its loss could trigger the intestinal inflammation [101]. We revealed that DSS administration resulted in a reduced alpha diversity of microbiota reflected by a decrease of OTU abundance (based on ACE and Chao1 indices). These results confirmed previous findings about dysbiosis and reduced microbiota diversity observed in UC [19,102,103]. The severity of IBD was inversely correlated with the microbial diversity indicating that the lower the microbial diversity, the higher the severity of IBD [104,105]. However, the precise causal relationship between the inflammatory state and a reduction in bacterial diversity remains unknown.

It was shown that the ratio between Firmicutes and Bacteroidetes during disease is changing and therefore is used as a characteristic marker of gut dysbiosis [106,107]. This study showed that Firmicutes were the most common phylum in the caecal microbiota of the DSS group, which was also confirmed by human studies [108]. Similar results were observed in a recent study [102], which also detected a considerable increase in Bacillaceae and Clostridiaceae in UC patients in relation to healthy controls. We have also confirmed lower abundance of Bacteroidetes in colitic rats with a significant decrease of *Bacteroidales bacterium*, *Bacteroides vulgatus* and *B. uniformis*. These bacteria are more prevalent in healthy patients [109], however, Conte et al. [110] found that among mucosa-associated bacteria, *B. vulgatus* presence was particularly low in patients with UC or CD. In the commensal microbiota *Bacteroides* predominate and provide many beneficial effects to the host including modulation of immune maturation [111]. On the other hand, *Bacteroides thetaiotaomicron* represented a significantly increased species in the DSS group of animals and induces colitis in rats [112]. Therefore, the observed expansion of *B. Thetaiotaomicron* could be the result of either severe inflammatory environment caused by DSS, or this species might represent one of the disease-inducing commensal bacterial strains. The study by Bloom et al. [113] confirmed that some commercial *Bacteroides* species were sufficient for IBD induction in antibiotic-pretreated mice, with *B. Thetaiotaomicron* as one of the most potent disease-inducing isolates. The phylum Verrucomicrobia was found to be less abundant in DSS animals compared to a healthy control group. Verrucomicrobia has been shown to induce regulatory immunity in mice and to have a positive impact on diseases modulated by inflammation [114,115]. Lachnospiraceae might also influence healthy functions, although different genera and species of this family increase in different diseases including IBD [116,117]. Their impact on the host physiology is often inconsistent across different studies. Our results confirmed that induction of colitis increased abundance of the family Lachnospiraceae, from which species *Roseburia* 499 and *Lachnospiraceae bacterium* 615 were significantly more prevalent in the DSS group compared to healthy controls. It has been reported that the number of *Escherichia coli* is elevated in UC, whether in mouse models or UC patients [118,119,120]. Moreover, *E.coli*, which was significantly increased in the colitic group, was considered as the reason for relapses in some patients with IBD [121].

In order to establish a comprehensive definition of microbial dysbiosis relevant to UC, microbiome data from many niches along the gastrointestinal tract is required. Therefore, we also observed the microbiomes of the caecum. We have shown that the bacterial communities of the faecal and the social content share the same major phyla-Firmicutes, Bacteroidetes and Actinobacteria, although there are significant differences at the genus and species level. Our results correlate with studies in animal models and humans [122,123]. The caeca of the DSS group have been found to harbour more clustered in comparison to faeces. It has been shown that some *Clostridium* species may play a significant role in the clinical course of IBD [124]. The benefits of *Lactobacillus* are well-known, and caecal samples in this study have shown high diversity in this genus and indicated potential for further probiotic research. The genus *Faecalibacterium* belongs to a butyrate producing bacteria also dominated in the charcoal samples [125]. These results suggest that the faecal microbial ecosystem was not representative of the caecum. Due to this fact, it is necessary to consider the selection of the sampling environment according to the experimental focus.

In conclusion, this study confirmed the advantages of a DSS colitis rat model such as its simplicity, reproducibility, and controllability. Moreover, our results suggest that the DSS model can provide additional information on the effect of specific treatments on different extraintestinal organ impairments. As gut microbiota was demonstrated to be an essential factor in pathogenesis of UC, detailed evaluation of microbial changes during the course of the disease represents the biggest benefit of this study. The observation that certain bacterial species in the gut microbiota are associated with UC raises the question of whether these organisms are the causal agents of contributors to, or a consequence of the disease. However, we need to mention that this model has some limitations. Since we used an animal model, our results cannot be directly transferred to the population of humans, although according to some literature the DSS model is considered as the most relevant model for the translation of rodent data to human disease [126]. The second limitation is associated with plasma CRP, which cannot be considered as a direct marker of inflammatory conditions in rats. Finally, in comparison to a chronic model of UC, the model of acute type of UC has the inflammatory stimulus removed after the induction period. This might be responsible for partial regeneration of gut mucosa in experimental animals. Despite that we observed this regeneration process in some parameters, the results from NGS analysis confirmed persistent dysbiosis of gut microbiota even five days after DSS removal. Since the colonic mucus-associated microbiota is even more closely correlated with disease severity than alterations in the faecal/caecal microbiota, it would be desirable to expand our results for NGS analysis of colon mucosa. Nevertheless, we confirmed the suitability of this model to monitor microbial changes during acute colitis, in order to test new attractive microbiome-based therapies. However, insights into the ecology of individual species will be essential if we want to understand the significance and consequences of differences in the gut microbiome.

## Figures and Tables

**Table 1:** Monoclonal antibodies used for flow cytometric analysis.

Markers	Conjugate	Isotype	Reactivity	Clone
CD3	PE	Mouse BALB/c IgG_1_, κ	Rat	G4.18
CD4	BV421	Mouse BALB/c IgG_1_, κ	Rat	OX-35
CD8α	BB700	Mouse BALB/c IgG_1_, κ	Rat	OX-8
CD11b	APC	Mouse BALB/c IgG_1_, κ	Rat	WT.5
CD45	APC-Cy7	Mouse BALB/c IgG_1_, κ	Rat	OX-1
Granulocytes	FITC	Mouse IgM, κ	Rat	HIS48

**Table 2:** Effect of dextran sulphate sodium (DSS) on body weight gain, food efficiency ratio, and food intake.

	DSS	C	*p*-Value
Body Weight Gain (g)	76.34 ± 7.54	96.33 ± 4.50	0.035
FER (%)	25.06 ± 2.51	32.86 ± 0.62	0.008
Food Intake (g/rat/day)	24.98 ± 0.45	24.80 ± 0.38	0.760

All values are expressed as mean ± SEM (*n* = 16/DSS group; *n* = 6/C group). FER (food efficiency ratio) (%) = (body-weight gain/food intake) × 100. DSS, induced colitis; C, healthy control.

**Table 3:** Evaluation of the lymphoid aggregates and follicles.

	DSS	C	*p*-Value
Lymphoid Aggregates (LA) per Rat	1.57 ± 0.61	0.33 ± 0.33	0.119
Lymphoid Follicles per Rat	5.00 ± 2.27	0.33 ± 0.33	0.088
Mean Length of LA (μm)	2495.00 ± 382.00	150.00 ± 150.00	<0.001
Mean Width of LA (μm)	481.80 ± 67.80	66.70 ± 66.70	0.005

Values are expressed as mean ± SEM (*n* = 16/DSS group; *n* = 6/C group). DSS, induced colitis; C, healthy control.

**Table 4:** Haematological parameters.

	1^st^ Collection		2^nd^ Collection		3^rd^ Collection	
	**DSS**	**C**	**DSS**	**C**	**DSS**	**C**
WBC (10^9^/L)	14.52 ± 0.53 ^α^	15.93 ± 1.03	22.37 ± 0.52 *^β^	18.52 ± 0.93	20.08 ± 1.36 ^β^	18.88 ± 0.90
LYM (10^9^/L)	10.97 ± 0.37 ^α^	12.66 ± 0.86	15.69 ± 0.44 *^β^	13.13 ± 0.97	15.37 ± 1.19 ^β^	14.74 ± 0.94
MON (10^9^/L)	0.50 ± 0.05 ^A^	0.45 ± 0.04	1.07 ± 0.07 *^B^	0.63 ± 0.13	0.83 ± 0.12 ^B^	0.78 ± 0.15
GRA (10^9^/L)	3.10 ± 0.16 ^α^	3.53 ± 0.34	5.03 ± 0.21 *^β^	3.62 ± 0.53	4.64 ± 0.45 ^β^	4.58 ± 0.42
HGB (g/L)	131.32 ± 2.25 ^α^	132.38 ± 3.19 ^γ^	145.57 ± 1.17 ^β^	150.00 ± 1.86 ^δ^	142.20 ± 1.08 ^β^	150.6 ± 3.39 ^δ^
RBC (10^12^/L)	5.61 ± 0.10 ^α^	5.65 ± 0.12 ^γ^	6.43 ± 0.09 ^β^	6.59 ± 0.09 ^δ^	6.29 ± 0.12 *^β^	6.77 ± 0.14 ^δ^
PLT (10^9^/L)	488.60 ± 24.90	431.50 ± 45.00 ^c^	513.70 ± 29.20 *	633.80 ± 41.10 ^d^	521.40 ± 85.70	488.6 ± 61.9 ^cd^
PLR	41.98 ± 1.95 ^α^	34.52 ± 3.52	34.69 ± 2.89 ^β^	44.64 ± 5.04	36.44 ± 4.36 ^αβ^	34.25 ± 5.76
LMR	27.32 ± 1.72 ^α^	29.10 ± 2.15	17.98 ± 1.14 *^β^	30.13 ± 2.46	18.48 ± 1.85 ^β^	21.04 ± 3.26
GLR	0.28 ± 0.01 ^α^	0.28 ± 0.02	0.32 ± 0.01 ^β^	0.28 ± 0.03	0.32 ± 0.03 ^β^	0.31 ± 0.01

Values are expressed as mean ± SEM (*n* = 16/DSS group; *n* = 6/C group). Means with the same letter or without symbol are not significantly different; different letters indicate significant difference between collection points, where *p* < 0.01 (A,B); *p* < 0.001 (α,β) for DSS group and *p* < 0.05 (c,d); *p* < 0.001 (γ,δ) for the control group. Symbols indicate significant differences between groups in the same collection point, where * *p* < 0.05. Abbreviations: WBC, white blood cells; LYM, lymphocytes; MON, monocytes; GRA, granulocytes; HGB, haemoglobin; RBC, red blood cells; PLT, platelets; PLR, platelet to lymphocyte ratio; LMR, lymphocyte to monocyte ratio; GLR, granulocyte to lymphocyte ratio. DSS, induced colitis; C, healthy control. 1^st^ collection, before induction of colitis (day 1); 2^nd^ collection, at the end of DSS administration (day 7); 3^rd^ collection, at the end of experiment (day 13).

## Data Availability

All data are incorporated into the article and its online Supplementary Material.

## Supplementary Materials

The following supporting information can be downloaded at: https://www.mdpi.com/article/10.3390/vetsci9050238/s1. Figure S1: Representative flow cytometry gating strategy for identification of T-cell subsets and neutrophils in the blood; Figure S2: Representative flow cytometry gating strategy for identification of T-cell subsets and neutrophils in the colon tissue; Figure S3: The cladogram plotted from LEfSe analysis showing tax enriched in the DSS (red) and control (green) groups; Figure S4: Venn diagrams for overlap of observed operational taxonomic units (OTUs) in faecal samples within DSS group collected at three different time points; Table S1: Levels of selected cytokines in colon tissue.
