# Supplementary material for: Dextran Sulphate Sodium Acute Colitis Rat Model: A Suitable Tool for Advancing Our Understanding of Immune and Microbial Mechanisms in the Pathogenesis of Inflammatory Bowel Disease
Source: Vet Sci. 2022 May 16;9(5):238. doi: 10.3390/vetsci9050238 (PMC9147231; doi:10.3390/vetsci9050238)
Supplement: Supplementary file 1 [file vetsci-09-00238-s001.zip › vetsci-1683865-supplementary.pdf]

Table S1. Levels of selected cytokines in colon tissue.

	Cytokine	Group	
		**DSS**	**C**
**pg/mg of tissue protein**	**IL-1α**	0.467±0.108	0.245±0.219
	**IL-1β**	132.80±44.1	11.08±0.727
	**IL-2**	0.362±0.049	0.145±0.072
	**IL-4**	0.022±0.006	0.023±0.009
	**IL-5**	0.583±0.096	0.261±0.081
	**IL-6**	0.026±0.006	0.028±0.008
	**IL-10**	0.628±0.138	0.699±0.350
	**IL-12p70**	3.892±0.861	1.260±0.179
	**IL-13**	0.179±0.062	0.422±0.394
	**G-CSF**	1.742±0.862	0.518±0.499
	**TNF-α**	0.857±0.546	0.243±0.102

Values are expressed as mean ± SEM (*n = 16/DSS group; n = 6/C group*). Abbreviations: IL, interleukin; G-CSF, granulocyte colony-stimulating factor; TNF-α, tumour necrosis factor α. DSS, induced colitis; C, healthy control.

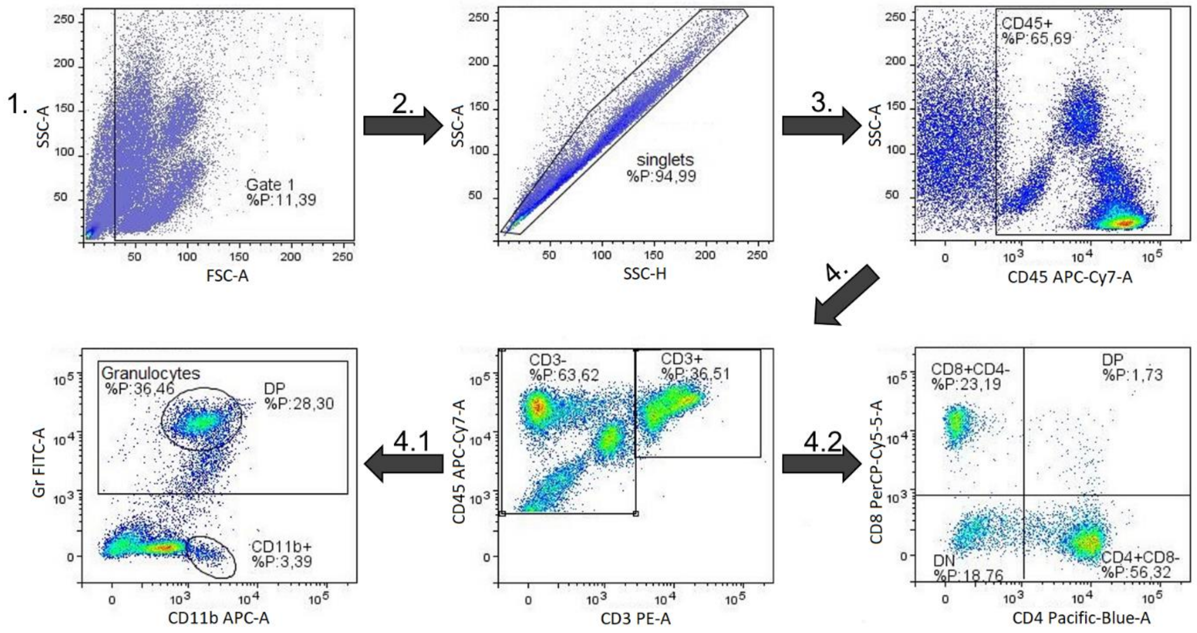

Figure S1. Representative flow cytometry gating strategy for identification of T-cell subsets and neutrophils in the blood.

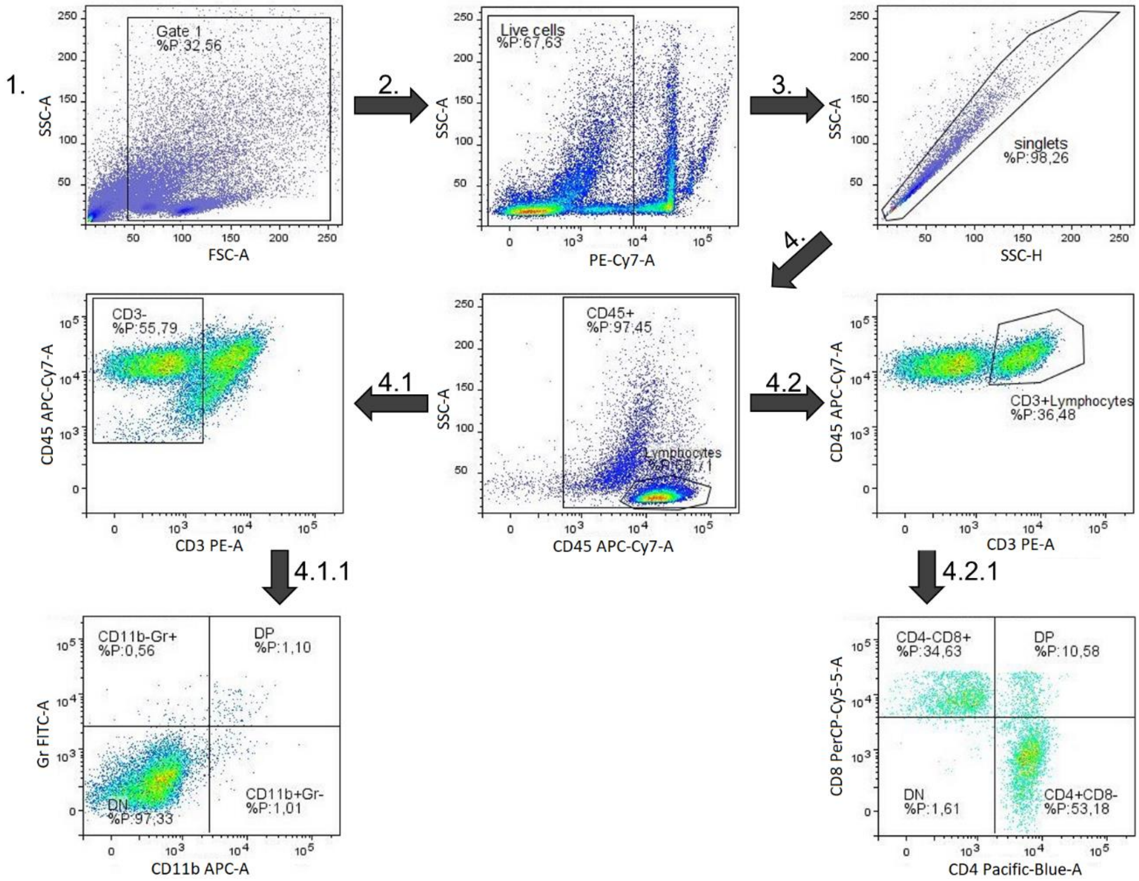

Figure S2. Representative flow cytometry gating strategy for identification of T-cell subsets and neutrophils in the colon tissue.

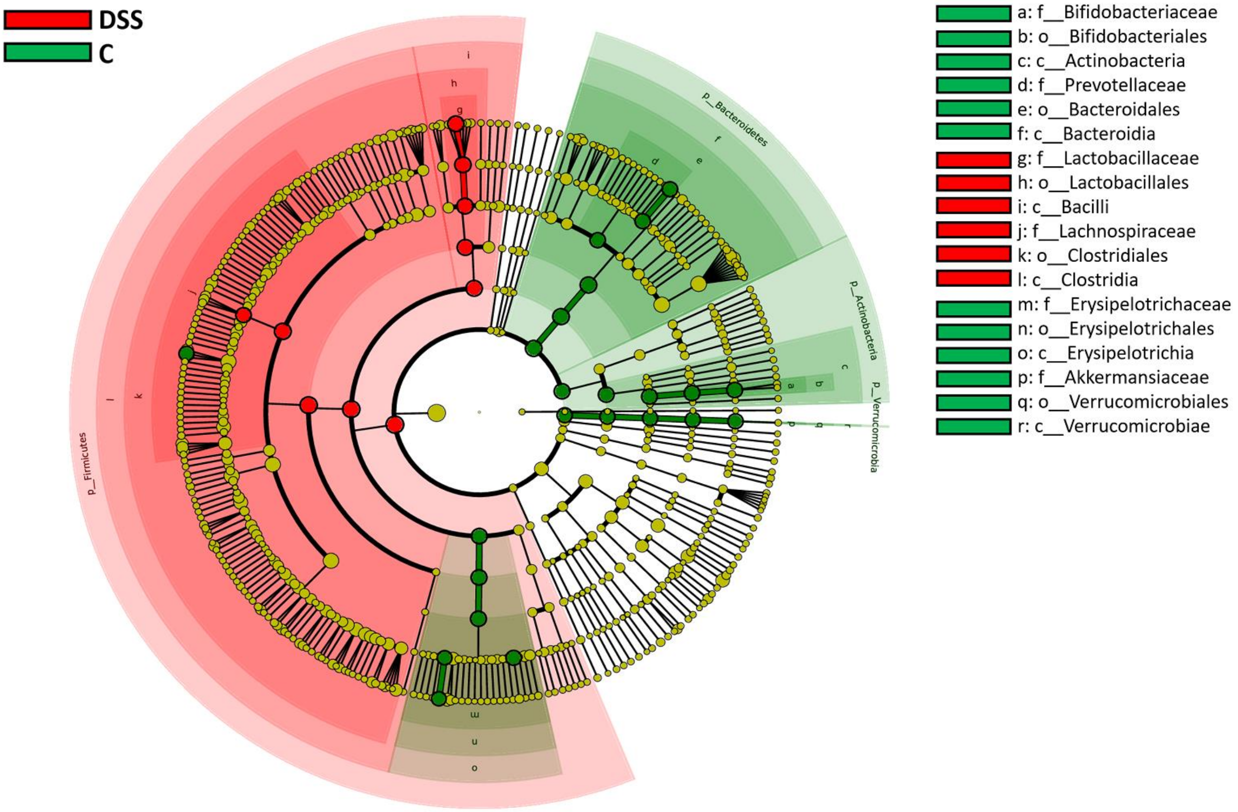

Figure S3. The cladogram plotted from LEfSe analysis showing taxa enriched in the DSS (red) and control (green) groups. The red and green circles mean that DSS and C showed differences in relative abundance and yellow circles mean non-significant differences. The name of the taxon level is abbreviated as c-class; o-order and f-family. DSS, induced colitis; C, healthy control.

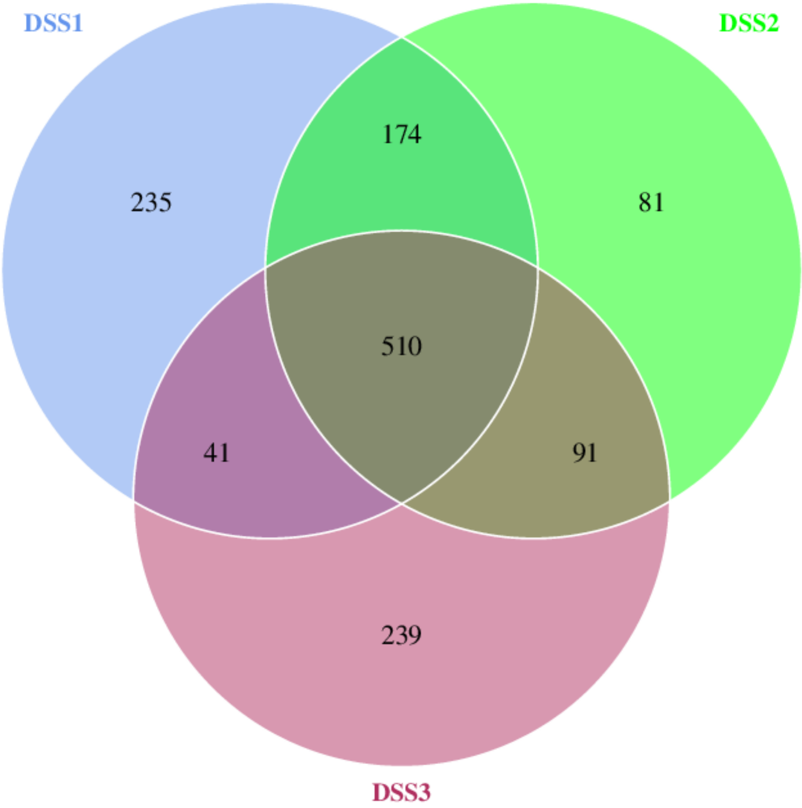

Figure S4. Venn diagrams for overlap of observed operational taxonomic units (OTUs) in faecal samples within DSS group collected at three different time points: 1, before induction of colitis (day 1); 2, at the end of DSS administration (day 7); 3, at the end of experiment (day 13). DSS, induced colitis.
